# Individual SMR neurofeedback responsiveness during a single session and its associations with autonomic regulation and shooting performance in elite rifle athletes

**DOI:** 10.3389/fnhum.2026.1922593

**Published:** 2026-09-04

**Authors:** Yan An, Zhengning Feng, Jiaojiao Lu

**Affiliations:** Shanghai Research Institute of Sports Science (Shanghai Anti-Doping Agency), Shanghai, China

**Keywords:** autonomic regulation, individual differences, neurofeedback responsiveness, neurofeedback training, rifle shooting, sensorimotor rhythm

## Abstract

**Introduction:**

Sensorimotor rhythm (SMR) neurofeedback has attracted growing interest for improving performance in precision sports; however, inter-individual variability in responsiveness remains poorly understood, and its associations with autonomic regulation and shooting performance have not been examined within a single-session paradigm. Because a substantial proportion of trainees show limited ability to self-regulate EEG activity (“non-responders”), characterizing responsiveness is important for developing personalized protocols. This exploratory study aimed to characterize individual differences in SMR neurofeedback responsiveness during a single session and examine their associations with post-training autonomic regulation and shooting performance in elite air rifle athletes.

**Methods:**

Thirteen elite air rifle shooters completed a single 10-min SMR (12–15 Hz) neurofeedback session at electrode site Cz. Heart rate variability (HRV) was recorded at four timepoints spanning pre- and post-shooting and pre- and post-training rest. Shooting performance was assessed via the SCATT system across two 30-shot blocks. Individual SMR regulation trajectories were indexed as linear amplitude slopes across five training blocks. Athletes were classified as Responders (slope > 0; *n* = 9) or Non-Responders (slope ≤0; *n* = 4) reflecting whether SMR amplitude showed a net increase or decrease across training.

**Results:**

SMR slope showed the strongest associations with changes in shooting score (r = 0.623) and logLF (r = −0.636), though neither survived Benjamini–Hochberg FDR correction (both q = 0.248). Responders exhibited larger post-training increases in DFA α2 (a long-term scaling exponent derived from detrended fluctuation analysis reflecting autonomic regulatory complexity; d = 1.688, *p* = 0.017) and larger decreases in logLF (d = 1.654, *p* = 0.019) than Non-Responders, though neither survived FDR correction. No baseline HRV (all *p* > 0.09) or subjective engagement (all *p* > 0.21) differences were observed between groups.

**Discussion:**

These preliminary, exploratory findings suggest that individual SMR regulatory capacity may relate to post-training autonomic modulation and shooting performance, and that SMR amplitude slope may represent a promising index of neurofeedback learning. Given the small and unequal responder groups (*n* = 9 vs. 4) and the absence of a sham or control condition, these associations should be interpreted as hypothesis-generating rather than confirmatory, warranting replication in larger, controlled samples.

## 1 Introduction

Precision shooting sports place exceptionally high demands on physical stability, fine motor coordination, and rapid decision-making (Ihalainen et al., 2016; Spancken et al., 2021). In the Olympic 10-meter air rifle event, for example, athletes must complete 60 shots within 75 min while aiming at a target whose 10-ring measures only 0.5 mm in diameter, leaving virtually no margin for error. Consequently, optimal performance depends not only on technical proficiency and physiological control but also on stable attentional focus and effective emotional regulation. Consistent with this view, previous research has identified stability, aiming accuracy, and trigger control as the key technical determinants of shooting performance, all of which are closely associated with athletes’ psychological state (Moreira da Silva et al., 2021; Lang and Zhou, 2022; Spancken et al., 2021; Sade et al., 1990). Moreover, competitive state anxiety has been shown to be negatively associated with shooting performance, highlighting the detrimental impact of momentary emotional fluctuations on competitive outcomes (Zhou et al., 2025; Zhao et al., 2024). However, athletes exhibit substantial inter-individual differences in psychophysiological regulatory responses even under identical training conditions, suggesting that standardized psychological interventions may not adequately address individual regulatory needs. Developing effective approaches to enhance athletes’ autonomous psychophysiological self-regulation under competitive pressure therefore remains an important challenge in sport psychology.

Psychological interventions such as imagery, self-talk, mindfulness, relaxation training, and biofeedback have been widely applied to optimize athletes’ psychological states and performance, with meta-analytic evidence supporting small-to-moderate benefits (Reinebo et al., 2023; Bühlmayer et al., 2017; Makaracı et al., 2024). Compared with conventional approaches relying primarily on cognitive strategies or physiological feedback, Neurofeedback Training (NFT) provides real-time feedback of electroencephalographic (EEG) activity, enabling individuals to voluntarily regulate specific brain oscillations through an operant-conditioning-like learning process. By directly targeting central nervous system activity, NFT may facilitate self-regulation while enhancing cognitive control and stress management (Cheng et al., 2024; Gruzelier, 2014a; Corrado et al., 2024). However, the effects of NFT on functional and performance outcomes remain inconsistent. For example, a randomized controlled trial in golfers found that successful modulation of frontal high-alpha power did not result in superior putting performance compared with controls (Ring et al., 2015), suggesting that EEG self-regulation capacity may not uniformly translate into performance enhancement and that individual variability in neurofeedback responsiveness warrants further investigation.

Among the various frequency bands of interest, the Sensorimotor Rhythm (SMR, 12–15 Hz) has received particular attention due to its association with cortical inhibition, sensorimotor decoupling, and motor preparation, making it a potential neurophysiological marker of fine motor control. Previous studies have demonstrated promising effects of SMR-NFT on motor performance and psychological regulation. For example, SMR training improved putting performance and enhanced SMR amplitude in golfers (Cheng et al., 2015), while beneficial effects on shooting stability, accuracy, and competitive psychological states have also been reported in precision athletes (Gong et al., 2020; Paul et al., 2012; Mutang et al., 2023). However, the magnitude of SMR modulation and its translation into performance benefits may depend on training protocols, task characteristics, and individual neurofeedback learning capacity (Afrash et al., 2023). These findings suggest that athletes may differ substantially in their ability to regulate SMR activity during neurofeedback training, highlighting the need to characterize individual variability in SMR learning trajectories.

Despite the considerable promise demonstrated by SMR neurofeedback training, existing research broadly confronts two core methodological challenges. First, inter-individual variability in training outcomes is substantial: approximately 15–30% of participants have been identified as “non-responders” who are unable to effectively regulate their EEG activity through neurofeedback (Oblak et al., 2017, 2019). When evaluating neurofeedback efficacy, the prevailing approach in the literature relies on pre-to-post mean comparisons or within-group trend tests at the aggregate level. These methods may have limited statistical power at the group level, particularly when sample sizes are small and individual modulation responses show substantial variability or heterogeneity in direction. Under such conditions, opposing individual response patterns may attenuate mean-level effects, potentially obscuring meaningful inter-individual differences in regulatory capacity. Using the slope of EEG amplitude time-series change during training as a dynamic index of individual modulation trajectory offers an effective means of identifying individuals with differential regulatory responsiveness, particularly when mean-level tests lack sufficient statistical power. This approach has emerged as a methodological trend in single-session neurofeedback sensitivity research (Bouny et al., 2022; Autenrieth et al., 2020). The present study is grounded in this rationale: on the basis of confirming limited statistical power for detecting group-level SMR amplitude trends, we introduce the SMR amplitude slope as a core dynamic indicator of individual regulatory responsiveness, with the aim of more precisely capturing athletes’ neural modulation trajectories during a single training session and their associations with psychophysiological and behavioral changes.

Second, the majority of existing SMR neurofeedback studies have focused predominantly on EEG activity or behavioral performance outcomes, with comparatively little attention paid to the relationship between SMR modulation trajectories and changes in autonomic nervous system (ANS) state. Heart rate variability (HRV), as an important physiological index for assessing ANS balance and autonomous regulatory capacity, has been demonstrated to be associated with neurofeedback training outcomes (Domingos et al., 2021; Mathew et al., 2025), and is widely used to index training stress and physiological readiness in athletic populations more broadly (Kayacan et al., 2023). HRV comprises multiple indices across time, frequency, and nonlinear domains, each reflecting distinct aspects of autonomic regulation. In particular, nonlinear measures such as detrended fluctuation analysis α2 (DFA α2) provide complementary information by characterizing complex temporal correlations within R-R interval dynamics beyond conventional HRV indices (Peng et al., 1995; Tulppo et al., 2005). Nevertheless, in the domain of precision shooting, the associations among SMR modulation trajectories, HRV alterations, and competitive performance remain insufficiently explored. In particular, individual differences in SMR regulatory capacity and their relationships with autonomic regulation and performance outcomes have yet to be directly investigated.

Grounded in these gaps, the present study employed a single-session SMR neurofeedback training paradigm to examine individual differences in regulatory responsiveness and their downstream consequences. Specifically, we addressed three questions: (1) How do individual SMR modulation trajectories vary during a single training session, and what degree of heterogeneity is present? (2) Does the SMR amplitude slope predict the magnitude of post-training changes in HRV and shooting performance? (3) Do athletes who differ in SMR regulatory responsiveness show distinct autonomic and performance outcomes following training?

To address these questions, we integrated SMR amplitude slope during neurofeedback training with multi-timepoint HRV monitoring and shooting performance assessment—a combination that, to our knowledge, has not been applied together in precision sport research. A four-timepoint HRV design was adopted specifically to separate the physiological effects of shooting-induced stress from those associated with the neurofeedback session itself. By characterizing SMR “responders” and “non-responders,” the study also aimed to generate preliminary evidence relevant to the design of individualized neurofeedback protocols. Given the single-group pre-post design, findings should be interpreted as associational; causal inference awaits studies with appropriate control conditions.

Based on this rationale, we hypothesized that: (1) SMR amplitude slopes during the single neurofeedback session would exhibit substantial inter-individual variability, with some athletes showing limited or absent positive SMR regulation trends; (2) greater SMR upregulation capacity, reflected by more positive SMR amplitude slopes, would be associated with more favorable post-training changes in HRV indices and shooting performance; and (3) athletes classified as Responders would exhibit distinct autonomic and behavioral response patterns compared with Non-Responders. Given the exploratory nature of this single-group study and the modest sample size, these hypotheses were considered directional expectations intended to guide interpretation rather than confirmatory predictions requiring definitive statistical verification.

## 2 Materials and methods

### 2.1 Participants

Fourteen elite rifle shooters were initially recruited (8 males, 6 females; mean age 17.79 ± 3.61 years; mean training experience 6.43 ± 2.03 years). All participants were registered competitive athletes currently engaged in systematic training. Athletic performance was classified according to the Chinese National Athletic Grading System (General Administration of Sport of China), which comprises five tiers from Class-III Athlete to International Master Athlete. The cohort included 2 International Master Athletes, 2 Master Athletes, and 10 Class-I Athletes. All participants were right-handed, had normal or corrected-to-normal vision, reported no history of neurological or psychiatric disorders, and had not experienced any major negative life events in the week prior to the experiment. One Class-1 Athlete was excluded from all analyses due to incomplete EEG recording during the fifth training block, which precluded the computation of a complete SMR amplitude time series. The final analytic sample comprised 13 participants (7 males, 6 females).

Sample size was estimated *a priori* using G*Power 3.1.9.7 (Faul et al., 2009). For a one-way repeated measures ANOVA (5 within-subject blocks), parameters were set at a medium effect size (*f* = 0.25), *α* = 0.05, power (1 − *β*) = 0.80, repeated-measures correlation r = 0.50, and nonsphericity correction *ε* = 1, yielding a minimum required sample of 12. The final sample of 13 satisfied this requirement. This power calculation applies specifically to the group-level repeated-measures analysis of SMR amplitude; power considerations for the correlational and subgroup analyses reported in Section 3.4–3.5 are discussed separately in the Limitations.

All participants provided written informed consent prior to the experiment; parental or guardian consent was obtained for those under 18 years of age. The study was approved by the Ethics Committee of the Shanghai Research Institute of Sports Science (Shanghai Anti-Doping Agency) (approval no.: LLSC20230014) and conducted in accordance with the Declaration of Helsinki.

### 2.2 Research design and procedure

The present study employed a single-group, single-session design with repeated measurements within the session. All experimental procedures were conducted at a 10-meter air rifle shooting range. The experimental protocol comprised five sequential phases, as described below. Throughout the protocol, respiration was not monitored or standardized; athletes breathed naturally without respiratory pacing or instruction, in order to preserve the ecological validity of the training and shooting environment.

1. Initial Resting Recording. Participants sat quietly with eyes open for 5 min, during which HRV data were recorded to establish an initial resting-state baseline.
2. First Shooting Test. Participants completed 30 live-fire shots using a 10-meter air rifle, with target lane assignments randomized across participants. Shooting performance was recorded using the SCATT MX-02 optoelectronic training system (SCATT Electronics LLC, Russia; Bale and Wilkinson, 2023); full details of system setup and data acquisition are provided in the Instruments section below.
3. Pre-Training Neurophysiological Baseline Recording. Following the first shooting test, participants underwent a 2-min eyes-closed resting period immediately followed by a 5-min eyes-open resting period, during which EEG and HRV data were recorded, respectively. The experimenter selected a continuous 2-min segment from the 5-min eyes-open recording for analysis, computing the mean SMR amplitude (12–15 Hz) to serve as the individualized feedback threshold for subsequent neurofeedback training and as an index of pre-training neurophysiological state.
4. SMR Neurofeedback Training. Participants completed a single 10-min SMR neurofeedback training session, consisting of five consecutive 2-min blocks separated by 10-s rest intervals. Throughout the session, the system continuously monitored SMR amplitude and EMG amplitude in real time and delivered positive visual feedback, in the form of an on-screen animation, only when two criteria were simultaneously satisfied: (1) EMG amplitude remained below a fixed threshold of 10 μV, serving as a muscle-tension inhibit criterion, and (2) SMR amplitude exceeded the individualized threshold derived from the pre-training baseline. Feedback was withheld whenever either criterion was not met, ensuring that positive feedback reflected genuine SMR upregulation rather than concurrent muscular activity. Participants were encouraged to regulate their SMR amplitude above the individualized threshold while minimizing muscle tension. Participants were guided to employ attentional focus, relaxation, and motor imagery strategies to enhance regulatory efficiency. Immediately upon completion of the training session, a further 2-min eyes-closed and 5-min eyes-open resting recording was conducted to assess acute post-training neurophysiological changes.
5. Second Shooting Test and Final Resting Recording. Participants completed a second set of 30 live-fire shots using the same equipment and procedural protocol as the first shooting test to ensure comparability. Following the second shooting test, participants completed a final 5-min eyes-open resting recording, during which HRV data were collected as an index of post-intervention recovery state.

In total, HRV resting-state data were collected at four timepoints: T1 (initial rest, pre-shooting baseline); T2 (post-first-shooting / pre-neurofeedback-training rest); T3 (post-neurofeedback-training rest); and T4 (final rest, post-second-shooting). T1 data were used to characterize baseline physiological status and to examine whether the subsequently defined responder and non-responder subgroups differed at baseline. The T2-to-T3 change score (ΔHRVtraining) was adopted as the primary index of the acute physiological correlates of neurofeedback training, specifically to isolate training-related autonomic changes from the confounding influence of shooting-induced stress on the pre-training baseline. T4 data served as a reference index of overall recovery state following completion of the full experimental protocol.

The rationale for adopting a single-group, single-session design was twofold. First, conducting research with elite competitive athletes is often constrained by their demanding training and competition schedules. Implementing a sham-controlled or multi-session protocol would have substantially increased recruitment demands and reduced the achievable sample size within this population, making such designs impractical given the exploratory nature of the present study. Second, the primary objective was to characterize inter-individual differences in the capacity for SMR self-regulation, operationalized as the within-session SMR amplitude slope, rather than to establish the efficacy of SMR-NFT relative to a sham condition. Accordingly, a within-subject slope-based approach was considered an appropriate and informative first step for characterizing individual differences in SMR self-regulation. This approach is consistent with previous single-session neurofeedback studies examining individual differences in regulatory responsiveness (Bouny et al., 2022; Autenrieth et al., 2020).

### 2.3 Instruments and equipment

#### 2.3.1 Neurofeedback training system

SMR neurofeedback training and EEG data acquisition were conducted using the Nexus-10 biofeedback system (Mind Media B.V., Roermond, The Netherlands), operated via the BioTrace+ software platform. The system supports real-time EEG signal acquisition and feedback delivery, making it suitable for neurofeedback training research. EEG signals were recorded via two available channels (A and B); Channel A was selected for the present study. The target training frequency band was the sensorimotor rhythm (SMR, 12–15 Hz). Electrode placement followed the international 10–20 system: the active training electrode was positioned at Cz (vertex), with the reference electrode placed at the left mastoid and the ground electrode at the right mastoid. EEG data were recorded continuously at a sampling rate of 256 Hz throughout the training session, with simultaneous generation of a real-time animated visual feedback interface through which participants could monitor and regulate their attentional state and cortical activity.

#### 2.3.2 Shooting performance assessment system

Shooting performance was assessed using the SCATT system, which records the dynamic aiming point trajectory during the aiming phase through to trigger release during live-fire trials. The system captures continuous kinematic indices of the shooting process, including aiming trajectory, aiming point velocity, and trigger release behavior. For all trajectory-based indices — including the percentage of time the aiming point remained within specified scoring zones — data from the 1-s window immediately preceding each trigger release were used for analysis (Lang and Zhou, 2022).

Prior to each shooting session, the SCATT MX-02 sensor was mounted at the anterior end of the rifle barrel using the V-shaped prism provided with the device. The sensor distance was set to 10 m in accordance with calibration requirements (Bale and Wilkinson, 2023). Following software initialization and entry of participant information, dry-fire and live-fire zero-point calibrations were performed sequentially to ensure the accuracy of trajectory data acquisition. Each participant then completed 30 consecutive live-fire shots, during which the system recorded real-time aiming trajectory and trigger release behavior. Upon completion, performance data were exported from the SCATT Expert software (Version 20.05.25; SCATT Electronics LLC, Russia) for subsequent analysis.

### 2.4 Outcome measures

#### 2.4.1 EEG indices

EEG data were recorded at the Cz electrode site within the SMR frequency band (12–15 Hz). Before each recording, electrode contact and EEG signal quality were checked through the BioTrace+ interface, and recording commenced only when the EEG/SMR signals were judged to be stable and free of obvious artifacts. Gross artifacts, such as movement-related fluctuations or electrode displacement, were monitored visually during the recording. Spectral analysis was performed using Fast Fourier Transform (FFT) with a window length of 2 s (corresponding to 512 sample points), a Hanning window function applied to minimize spectral leakage, and a window overlap of 75%, yielding spectral updates at 0.5-s intervals. For offline FFT-based spectral analysis, BioTrace+'s built-in artifact-rejection function was enabled, with the EMG artifact frequency range set to 75–100 Hz. The primary EEG metric was absolute amplitude (unit: μV), defined as the voltage amplitude within the target frequency band (12–15 Hz), reflecting the overall energy level of neural activity in that band.

To characterize each athlete’s individual modulation trajectory across the single training session, a linear slope was computed for each participant by regressing the SMR amplitude change scores — calculated as the difference between each block’s mean SMR amplitude and the pre-training baseline — against the sequential block number (Blocks 1 through 5). The resulting regression coefficient (slope) served as a dynamic index of the direction and magnitude of each individual’s SMR regulatory trend across the training session.

#### 2.4.2 HRV indices

HRV data were collected using the Polar H10 chest strap (Polar Electro Oy, Kempele, Finland). Participants self-applied the sensor, with the electrode surface secured firmly against the chest. R-R interval data were recorded and stored via the Polar Vantage V2 wrist monitor (Polar Electro Oy, Kempele, Finland), downloaded from the Polar Flow web platform, and imported into Kubios HRV Standard software (version 3.5.0; Kubios Oy, Kuopio, Finland; Tarvainen et al., 2014) for analysis. Artifact correction was applied using the threshold-based method to identify and correct ectopic beats and motion-related artifacts in the inter-beat interval series.

The following HRV indices were extracted. Time-domain indices: the standard deviation of normal-to-normal R-R intervals (SDNN), reflecting overall autonomic nervous system regulatory capacity; and the root mean square of successive differences in R-R intervals (RMSSD), reflecting vagally mediated short-term heart rate fluctuations (Shaffer and Ginsberg, 2017; Bouny et al., 2022). Frequency-domain indices: Low-Frequency power (LF; 0.04–0.15 Hz) and High-Frequency power (HF; 0.15–0.40 Hz) were each extracted in two representations: log-transformed values (logLF, logHF) to address positive skewness in the raw power distributions, and normalized units [LF (n.u.), HF (n.u.)] to assess sympathovagal balance independent of total power fluctuations (Shaffer and Ginsberg, 2017; Bouny et al., 2022). The LF/HF ratio was additionally computed as a composite index of sympathovagal balance. Total Power (TP; ms^2^) was retained as an index of overall autonomic activity. Composite autonomic indices: Stress Index, Parasympathetic Nervous System Index (PNS Index), and Sympathetic Nervous System Index (SNS Index), as computed by Kubios HRV Standard software. Nonlinear indices: Detrended Fluctuation Analysis scaling exponents DFA α1 (short-term fluctuations; scale range: 4–16 beats) and DFA α2 (long-term fluctuations; scale range: 16–64 beats), reflecting the fractal correlation properties of the R-R interval series across different timescales (Peng et al., 1995) (see Table 1).

**Table 1:** Description of HRV indices and their physiological interpretations.

Category	Index	Unit	Physiological Interpretation
Overview	Stress index	–	Reflects overall physiological stress level; higher values indicate stronger sympathetic activation
	PNS index	–	Reflects the relative level of parasympathetic activity and recovery capacity; higher values indicate greater vagal tone
	SNS index	–	Reflects sympathetic arousal level, indicating degree of tension and alertness
Time domain	RMSSD	ms	Reflects parasympathetic (vagal) cardiac modulation; higher values indicate greater vagal tone
	SDNN	ms	Reflects overall autonomic variability encompassing both sympathetic and parasympathetic contributions
Frequency domain	logLF	log (ms^2^)	Natural logarithm of LF power; reflects the overall sympathetic–parasympathetic regulatory tone
	LF (n.u.)	n.u.	Normalized LF power; primarily reflects sympathetic nervous system modulation
	logHF	log (ms^2^)	Natural logarithm of HF power; reflects vagal (parasympathetic) activity
	HF (n.u.)	n.u.	Normalized HF power; reflects vagal nervous system activity
	LF/HF	–	Traditionally interpreted as a sympathovagal balance index, though this interpretation has been challenged (Billman, 2013) and should be considered with caution
	Total Power (TP)	ms^2^	Reflects overall HRV energy; describes global autonomic nervous system activity
Nonlinear	DFA α_1_	–	Short-term fractal scaling exponent of the R-R interval series (scale: 4–16 beats); a value approximating 1.0 reflects healthy fractal correlation structure
	DFA α_2_	–	Long-term fractal scaling exponent of the R-R interval series (scale: 16–64 beats); reflects system-level long-range stability and the capacity to maintain autonomic homeostasis during sustained tasks

HRV indices were extracted using Kubios HRV Standard software (version 3.5.0; Kubios Oy, Kuopio, Finland). Abbreviations: LF, low frequency; HF, high frequency; n.u., normalized units; DFA, detrended fluctuation analysis.

#### 2.4.3 Sport performance indices

Ten raw performance variables exported from SCATT Expert software (version 20.05.25) were selected for data organization and statistical analysis. The indices are described in Table 2.

**Table 2:** Description of SCATT performance indices.

Variable	Description
Shot score	Mean shot score per trial, expressed on the ISSF air rifle scoring scale
Aim time (s)	Duration of continuous on-target aiming immediately preceding trigger release
10.0 (%)	Proportion of aiming time falling within the 10.0-ring zone during the 1-s pre-shot window
10.5 (%)	Proportion of aiming time falling within the 10.5-ring zone during the 1-s pre-shot window
10a0 (%)	Proportion of aiming time falling within a 10-ring zone centered on the mean aiming point during the 1-s pre-shot window
10a5 (%)	Proportion of aiming time falling within a 10.5-ring zone centered on the mean aiming point during the 1-s pre-shot window
S1 (mm s^−1^)	Mean velocity of the aiming point trajectory on the target face during the 1-s pre-shot window
S2 (mm s^−1^)	Mean velocity of the aiming point trajectory on the target face during the 250 ms pre-shot window
DA (mm)	Distance between the mean aiming point and the actual impact point during the 1-s pre-shot window, reflecting trigger release cleanlines

Variable descriptions adapted from Bale and Wilkinson (2023) and Lang and Zhou (2022).

### 2.5 Data processing and statistical analysis

Statistical analyses were performed using JASP (version 0.19.2.0; JASP Team, 2024, https://jasp-stats.org). All variables are reported as mean ± standard deviation (M ± SD).

#### 2.5.1 SMR dynamic trend analysis

The mean SMR amplitude across five training blocks was used as the primary observation variable. A one-way repeated-measures analysis of variance (RM-ANOVA) was conducted to examine overall differences across training stages, supplemented by a linear trend test. Mauchly’s test of sphericity was applied prior to analysis; where the sphericity assumption was violated, Greenhouse–Geisser corrected values are reported. To characterize individual regulatory trajectories, the change in SMR amplitude relative to each athlete’s individual baseline was calculated for each of the five blocks. For each participant, an individual SMR amplitude slope was computed using the SLOPE function in Microsoft Excel (Microsoft Corporation, Redmond, WA, United States), with block number (1–5) as the independent variable and the SMR amplitude change score (relative to individual baseline) as the dependent variable. The resulting slope served as an individual index of neurofeedback regulatory response across the session. A one-sample *t*-test against zero was subsequently performed to determine whether the distribution of slopes across all athletes differed significantly from zero.

#### 2.5.2 Responder classification

On this basis, athletes were classified as Responders (slope > 0) or Non-Responders (slope ≤ 0) according to the sign of their individual slope, reflecting whether a participant exhibited, on average, a training-consistent increase in SMR amplitude irrespective of its magnitude. This criterion is consistent with the dynamic responsiveness framework adopted by Bouny et al. (2022) and Autenrieth et al. (2020) in single-session neurofeedback sensitivity research. We note that this classification is *post hoc*, derived from the same within-session training data subsequently used to compare groups on autonomic and performance outcomes, which introduces a degree of circularity; this limitation is discussed further below (see Discussion, Limitations). Because a zero-based criterion is inherently sensitive to slopes close to zero, we additionally conducted a sensitivity analysis using the sample mean slope (M = 0.103) as an alternative, non-zero cut-point.

#### 2.5.3 Baseline comparability

Independent-samples t-tests were used to compare responders and non-responders on key HRV indices recorded at T1 (initial resting baseline) to verify that the two groups were comparable in initial physiological state.

#### 2.5.4 Change score computation

Change scores were calculated as ΔValue = Post − Pre. ΔHRVtraining was derived by subtracting T2 (pre-training resting HRV) from T3 (post-training resting HRV), capturing the acute physiological response attributable to the neurofeedback session. Δshooting performance was computed by subtracting the score of the first 30 shots from that of the last 30 shots.

#### 2.5.5 Supplementary linear mixed-effects analysis

**Figure 1:**
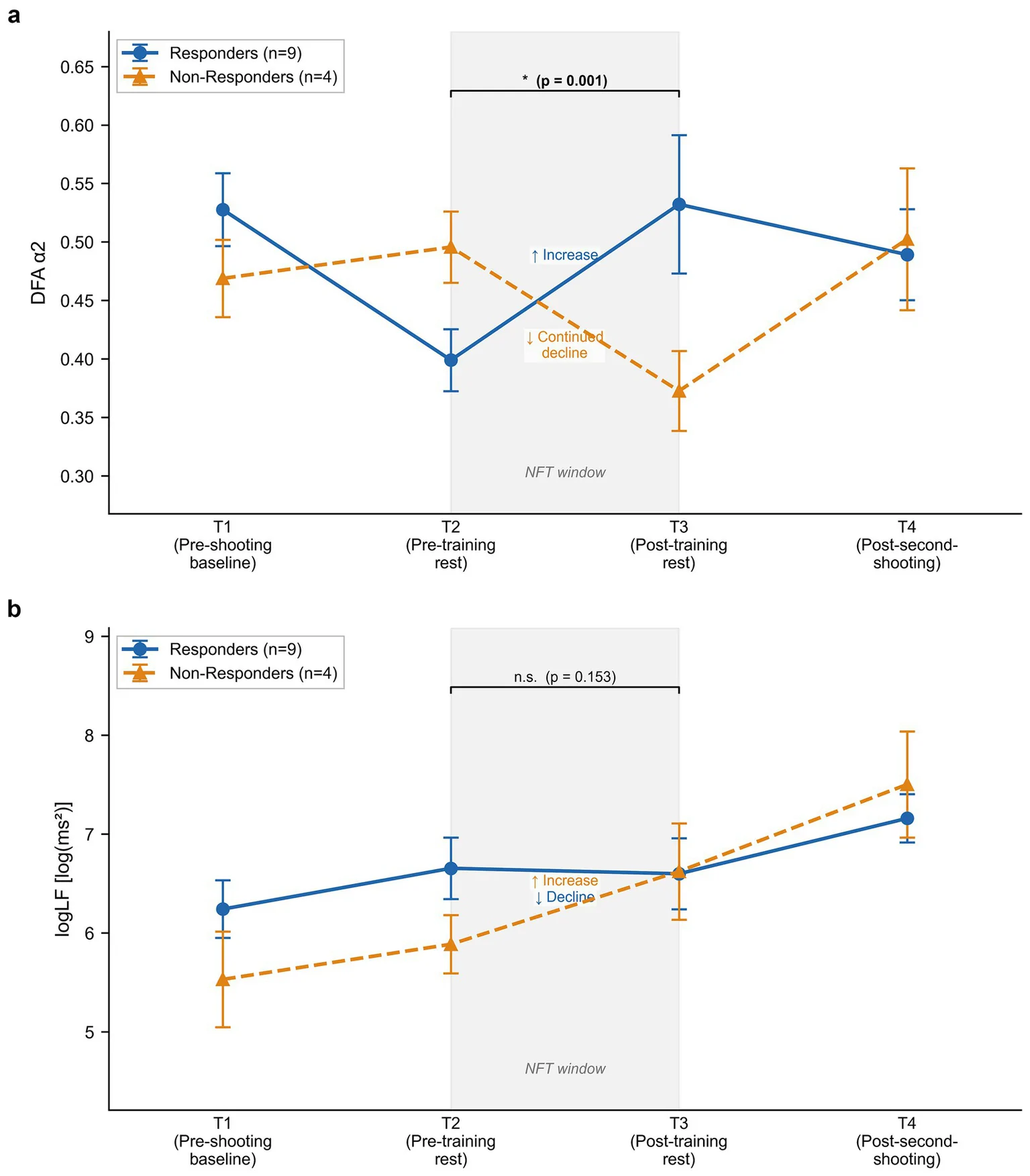
Temporal changes in autonomic indices in responders and non-responders. **(a)** Changes in DFA *α*2 across the four assessment time points (pre-shooting baseline, pre-training rest, post-training rest, and post-second shooting). **(b)** Changes in log*LF* across the same four assessment time points. Data are presented as mean ± SE. Shaded region indicates the NFT training window (T2–T3). Linear mixed-effects models indicated a significant Timepoint × Group interaction for DFA *α*2 (*p* = 0.010) but not for log*LF* (*p* = 0.093); see Supplementary Table S4.

To provide a robustness check for the longitudinal HRV trajectories across the four timepoints shown in Figure 1, linear mixed-effects models (LMMs) were additionally fitted for DFA α2 and logLF in JASP (version 0.19.2.0, Mixed Models module). Timepoint (T1–T4), Group (Responder/Non-Responder), and their interaction were specified as fixed effects, with a random intercept for participant. A random slope for Timepoint was initially considered but was not estimable given the sample size and was therefore removed from the final model. Fixed effects were evaluated using Type III likelihood-ratio tests. To isolate the planned between-group difference in the T2-to-T3 change, which was not directly available through JASP’s Mixed Models interface, the relevant model coefficients and variance–covariance matrix were exported and the corresponding linear contrast was computed in Python using statsmodels (version 0.14). The resulting contrast estimate was independently verified against the JASP model output.

#### 2.5.6 Primary analyses and multiple comparison correction

All HRV change indices and shooting performance change indices were examined in correlation analyses and between-group comparisons. Given the exploratory nature of the analyses, false discovery rate (FDR) correction was applied to all comparisons using the Benjamini–Hochberg procedure (q < 0.05). Effect sizes are reported throughout: Pearson r with 95% confidence intervals (CIs) for correlation analyses, and Cohen’s d with 95% CIs for t-tests.

#### 2.5.7 Control for confounding factors

To rule out motivational and effort-related confounds, athletes completed a brief post-training questionnaire consisting of five items rated on a 7-point Likert scale, assessing fatigue, tension, willingness to engage, effort exerted, and perceived difficulty. Independent-samples *t*-tests were used to compare responders and non-responders on each of these five dimensions.

## 3 Results

### 3.1 Dynamic changes in SMR amplitude across training blocks

**Figure 2:**
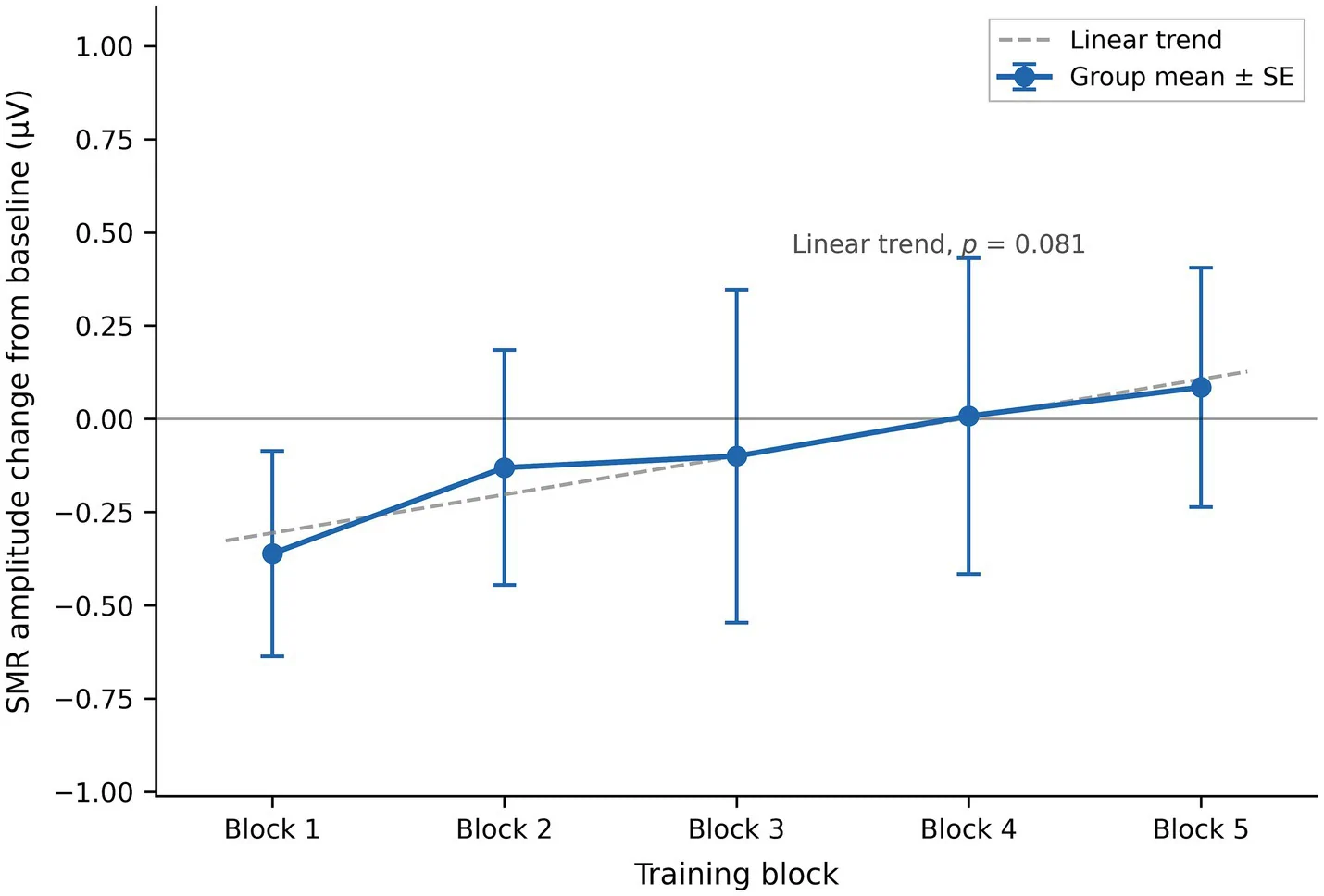
Changes in SMR amplitude across five neurofeedback training blocks. Mean SMR amplitude (12–15 Hz) recorded at Cz across five consecutive neurofeedback training blocks during a single training session. Data are presented as mean ± SE.

Mauchly’s test of sphericity indicated that the sphericity assumption was satisfied (*W* = 0.468, *p* = 0.548). The repeated-measures ANOVA revealed no significant main effect of training block on SMR amplitude (*F*(4,48) = 0.858, *p* = 0.496, η^2^ = 0.067; see Figure 2). A linear trend test, based on the pooled within-subject error term of the repeated-measures ANOVA, indicated a positive linear trend in SMR amplitude across training blocks, although this effect did not reach statistical significance (Estimate = 0.326, SE = 0.183, t(48) = 1.780, *p* = 0.081). The normalized linear contrast estimate (0.326) corresponded to the mean individual slope (0.103) after applying the orthonormal polynomial scaling factor (√10), indicating consistency in the underlying point estimates across the two approaches. This group-level trend was consistent with the individual-level slope analysis reported in Section 3.2, although the two approaches yielded different inferential results because they used different error terms and degrees of freedom.

### 3.2 Individual differences in SMR self-regulation trajectories

**Figure 3:**
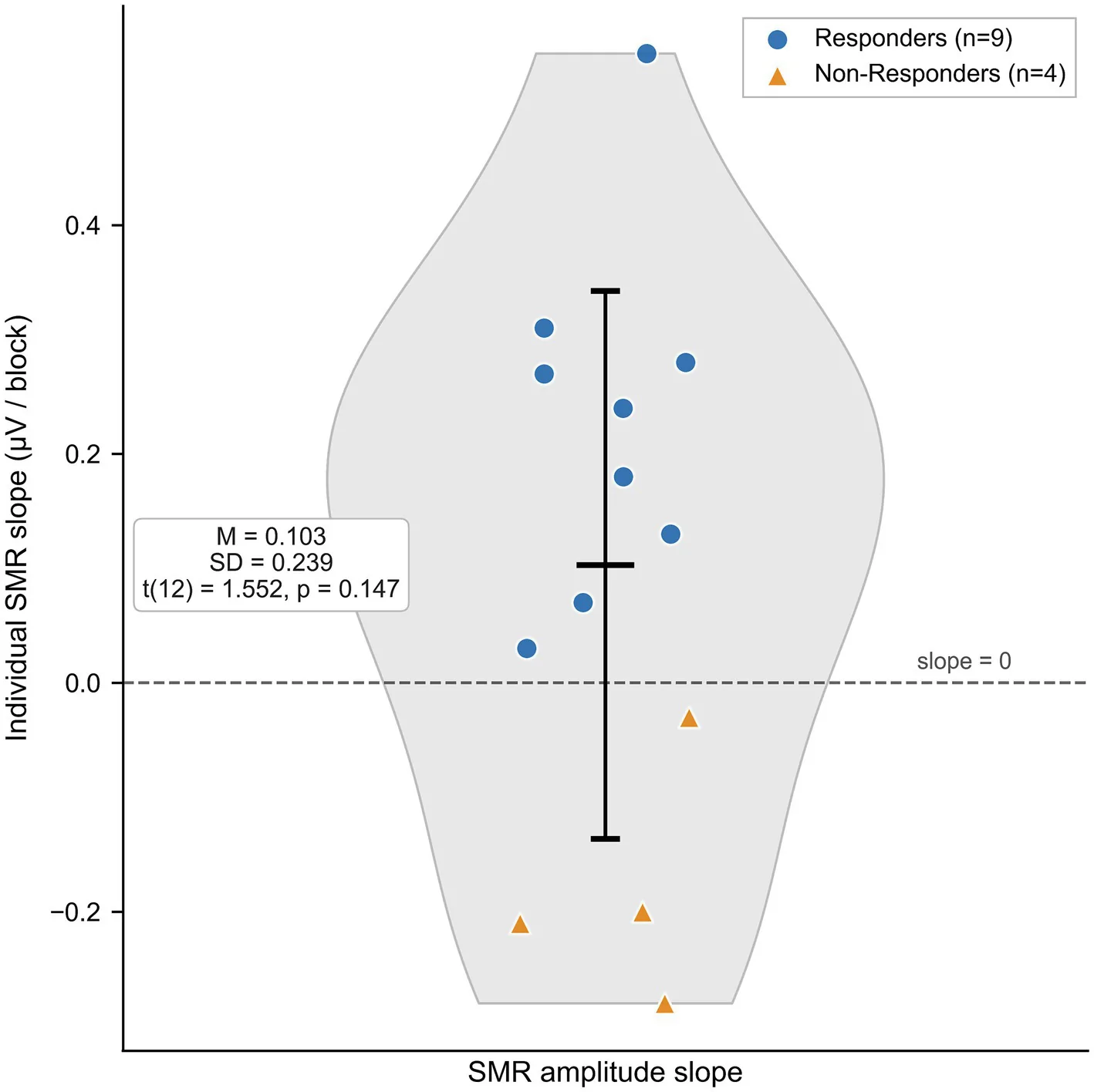
Distribution of individual SMR amplitude slope values. Violin plot showing the distribution of SMR amplitude slope values across the 13 athletes. The mean (*M*), standard deviation (*SD*), and results of the one-sample *t*-test against zero are displayed.

A one-sample *t*-test indicated that the mean SMR slope across the 13 athletes was 0.103 (SD = 0.239), which did not differ significantly from zero (t(12) = 1.552, *p* = 0.147). The slope distribution exhibited marked inter-individual heterogeneity: the standard deviation (0.239) substantially exceeded the mean (0.103), and individual slopes spanned both negative and positive values (see Figure 3). Based on the classification criterion (slope > 0), nine athletes were classified as responders (69.2%) and four as non-responders (30.8%), A sensitivity analysis using the sample mean slope M = 0.103 as an alternative cut-point reclassified two athletes whose individual slopes ranged from 0 to 0.103 from Responders to Non-Responders, resulting in seven Responders and six Non-Responders rather than the original nine Responders and four Non-Responders. The two primary between-group differences identified under the original classification remained consistent in direction and statistically significant under this alternative grouping (ΔDFA α2: t(11) = 2.293, *p* = 0.043, d = 1.276; ΔlogLF: t(11) = −2.211, *p* = 0.049, d = −1.230), though, as with the original classification, neither remained statistically significant after Benjamini–Hochberg FDR correction across the 22 comparisons (q = 0.361 for both). The consistency of findings across both classification schemes supports the robustness of the zero-slope criterion adopted as the primary classification approach in this study.

### 3.3 Baseline physiological comparability between responders and non-responders

Independent-samples t-tests were conducted on all HRV indices recorded at T1. No significant between-group differences were found for any of the indices examined (all *p* > 0.05; see Supplementary Table S1).

### 3.4 Correlations between SMR slope and outcome measures

Pearson correlation analyses were conducted between the SMR slope and all HRV change indices and shooting performance change indices, with the Benjamini–Hochberg FDR procedure applied to control for multiple comparisons (q < 0.05). The SMR slope showed the strongest associations with ΔR (shot score) (r = 0.623, 95%CI [0.110, 0.874], *p* = 0.023) and ΔlogLF (r = −0.636, 95% CI [−0.879, −0.131], *p* = 0.019); however, neither survived Benjamini–Hochberg FDR correction across the full set of 22 comparisons (q = 0.248 for both). ΔStress index (r = 0.590, *p* = 0.034), ΔDFA α2(r = 0.509, *p* = 0.076), and Δ10.5% (r = 0.500, *p* = 0.082) showed the next highest correlations; none reached significance after FDR correction. Complete correlation results are presented in Supplementary Table S2.

### 3.5 Between-group differences in HRV and shooting performance outcomes

Between-group comparisons were conducted on all HRV change indices and shooting performance change indices, with FDR correction applied (Benjamini–Hochberg procedure, *q* < 0.05).

**Figure 4:**
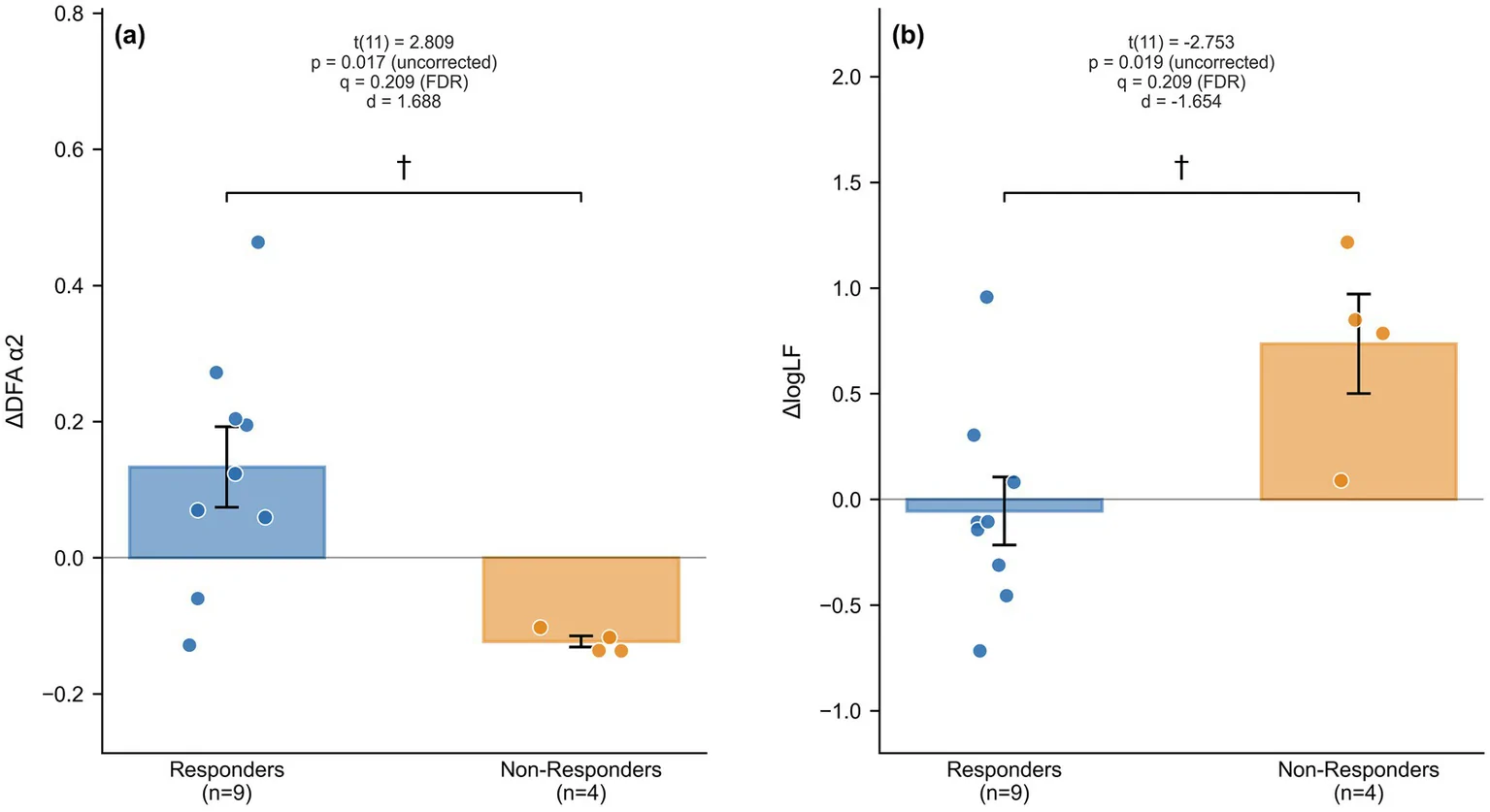
Comparison of changes in autonomic indices between responders and non-responders. **(a)** Change in detrended fluctuation analysis scaling exponent α2 (ΔDFA α2). **(b)** Change in log-transformed low-frequency power (ΔlogLF). Change scores reflect T3 minus T2. Data are presented as mean ± SE. Responders *n* = 9: slope > 0; Non-responders *n* = 4: slope ≤ 0. †*p* < 0.05 (uncorrected); neither comparison survived Benjamini–Hochberg FDR correction (see Supplementary Table S3).

Responders exhibited greater increase in ΔDFA α2 than non-responders (responders: 0.133 ± 0.178; non-responders: −0.123 ± 0.017; t(11) = 2.809, *p* = 0.017, d = 1.688, 95% CI [0.178, 3.198]) and greater reduction in ΔlogLF (responders: −0.055 ± 0.481; non-responders: 0.736 ± 0.471; t(11) = −2.753, *p* = 0.019, d = −1.654, 95% CI [−3.157, −0.151]); Although neither comparison remained significant after Benjamini–Hochberg FDR correction across the 22 between-group comparisons (q = 0.209 for both), these two measures showed the largest effect sizes among all autonomic indices examined (see Figure 4).

Regarding shooting performance, responders showed greater Δ10.5% (responders: 0.097 ± 0.101; non-responders: −0.024 ± 0.036; t(11) = 2.280, *p* = 0.044, d = 1.370, 95% CI [−0.079, 2.819]), Δ10.0% (responders: 0.076 ± 0.109; non-responders: −0.044 ± 0.042; t(11) = 2.084, *p* = 0.061, d = 1.252, 95% CI [−0.177, 2.681]), and ΔR (responders: 0.041 ± 0.147; non-responders: −0.197 ± 0.293; t(11) = 1.994, *p* = 0.071, d = 1.198, 95% CI [−0.222, 2.619]) than non-responders, all yielding large effect sizes (d = 1.198–1.370); however, none reached significance after FDR correction. Complete between-group results are presented in Supplementary Table S3.

Four-timepoint HRV trajectories are displayed in Figure 1. For DFA α2, responders showed a decline from T1 to T2 (0.528 ± 0.031 → 0.399 ± 0.026), a recovery from T2 to T3 (0.399 ± 0.026 → 0.532 ± 0.059), and a partial decline at T4 (0.489 ± 0.039). Non-responders showed a continued decline from T2 to T3 (0.496 ± 0.030 → 0.373 ± 0.034) followed by an increase at T4 (0.502 ± 0.061). For logLF, responders showed relatively stable values from T2 to T3 (6.653 ± 0.310 → 6.598 ± 0.360), while non-responders showed an increase over the same interval (5.885 ± 0.295 → 6.621 ± 0.487).

To formally test the divergent trajectories visualized in Figure 1, we additionally fitted linear mixed-effects models (LMM) for DFA α2 and logLF across all four timepoints (see Section 2.5). The Timepoint × Group interaction was significant for DFA α2 (likelihood ratio χ^2^(3) = 11.27, *p* = 0.010), and a planned contrast isolating the differential T2-to-T3 change between groups closely paralleled and further strengthened the corresponding change-score comparison reported above (estimate = 0.256, SE = 0.080, z = 3.20, *p* = 0.001). For logLF, the Timepoint × Group interaction did not reach significance χ^2^(3) = 6.42, *p* = 0.093, and the corresponding T2-to-T3 contrast was likewise non-significant (estimate = −0.791, SE = 0.553, z = −1.43, *p* = 0.153), indicating that this effect was more sensitive to the specific analytic approach than the DFA α2 finding. Full model output is reported in Supplementary Table S4.

### 3.6 Post-training subjective ratings

Independent-samples t-tests were conducted on five subjective rating dimensions assessed immediately after training. No significant between-group differences were found for any dimension: fatigue (responders: 3.22 ± 1.30; non-responders: 4.25 ± 1.26; t(11) = −1.326, *p* = 0.212), tension (responders: 2.11 ± 0.93; non-responders: 3.00 ± 1.83; t(11) = −1.194, *p* = 0.258), willingness to engage (responders: 5.67 ± 1.41; non-responders: 5.75 ± 1.26; t(11) = −0.101, *p* = 0.921), effort exerted (responders: 5.56 ± 1.13; non-responders: 5.50 ± 0.58; t(11) = 0.092, *p* = 0.929), and perceived difficulty (responders: 4.33 ± 1.00; non-responders: 5.00 ± 1.63; t(11) = −0.920, *p* = 0.377).

## 4 Discussion

This exploratory pilot study examined individual differences in SMR self-regulation trajectories during a single neurofeedback session in elite rifle athletes and their preliminary associations with post-training autonomic nervous system responses and shooting performance. Importantly, the principal correlational and between-group findings reported below did not survive correction for multiple comparisons and should therefore be interpreted as hypothesis-generating rather than confirmatory. At the group level, SMR amplitude did not show a significant main effect across training blocks, and the linear trend, while positive in direction, did not reach statistical significance (*p* = 0.081). This pattern is broadly consistent with prior neurofeedback research suggesting that inter-individual variability in the direction of neural regulation can attenuate group-mean effects (Gruzelier, 2014b; Alkoby et al., 2018). When individual SMR amplitude slopes were examined, marked heterogeneity was evident: some athletes exhibited sustained upward regulatory trends while others showed no systematic change or negative trajectories. The proportion of athletes classified as non-responders in the present sample (4 of 13, 30.8%) was comparable to previous reports (15–30%; Oblak et al., 2017, 2019), although this comparison should be interpreted cautiously given the small sample size. This heterogeneity became apparent only when individual slope trajectories were examined, whereas the group-level RM-ANOVA yielded a non-significant main effect, a pattern that would have remained hidden under conventional group-mean analysis alone. These observations tentatively suggest that conventional group-mean analyses may be less sensitive to inter-individual differences in neurofeedback responsiveness than analyses based on individual slope trajectories, and that individualized slope indices may provide a useful complementary approach for single-session neurofeedback research. This possibility warrants further investigation in larger, well-controlled studies.

The uncorrected associations between SMR slope and post-training changes in sympathetic activation and shooting performance suggest that the capacity for voluntary SMR upregulation during a single neurofeedback session may be related to subsequent psychophysiological state. However, these associations did not survive FDR correction and should therefore be regarded as preliminary. If replicated, the attenuation of sympathetic activation observed in athletes with stronger SMR upregulation trajectories would be consistent with previous evidence that reduced low-frequency autonomic activity is associated with greater physiological stability in precision sports contexts (Shaffer and Ginsberg, 2017; Ortega and Wang, 2018). A parallel, non-significant trend toward improved shooting performance was observed, although this change cannot be specifically attributed to SMR upregulation given the absence of a control or sham-feedback condition. Similar performance changes have been reported in professional golfers following a single SMR neurofeedback session (Wu et al., 2024); while this parallel is consistent with a possible role for SMR regulation in fine motor control across precision sports, the present single-group design cannot rule out practice or familiarization effects as alternative explanations for the shooting-performance changes observed here. Whether a comparable relationship exists in shooting sports, and whether SMR regulation contributes causally to such changes, remains to be determined in adequately powered, controlled studies. The uncorrected positive association between SMR slope and ΔStress Index (r = 0.590, *p* = 0.034) similarly did not survive FDR correction (q = 0.248) and should be interpreted with the same caution as the associations above. At face value, this pattern suggests that athletes with stronger SMR upregulation exhibited higher post-training Stress Index values, potentially reflecting the physiological cost of sustained attentional engagement during neurofeedback training, which could transiently delay sympathetic recovery after task completion. This pattern appears, at least descriptively, to run counter to the concurrent negative association with ΔlogLF; however, given that neither association survived correction for multiple comparisons, we are cautious about over-interpreting this apparent divergence. Stress Index and logLF capture different characteristics of the R–R interval series and therefore are not necessarily expected to show parallel changes over an acute, single-session timescale. Nonetheless, we cannot rule out the possibility that this divergence reflects sampling variability in a small, likely underpowered sample rather than a genuine physiological dissociation. It should also be noted that the physiological interpretation of LF power as a specific marker of cardiac sympathetic activity has been challenged; LF power is considered to reflect a complex mixture of sympathetic, parasympathetic, and other regulatory influences rather than sympathetic activity alone (Billman, 2013). The logLF findings reported here should therefore be interpreted as reflecting broader autonomic modulation rather than a specific attenuation of sympathetic activation.

Responders and non-responders showed descriptively divergent post-training autonomic profiles, though neither between-group comparison survived FDR correction and both should be interpreted with corresponding caution. The larger increase in DFA α2 observed in responders (d = 1.688, *p* = 0.017, uncorrected) is suggestive of stronger post-training long-term autonomic regulatory complexity in this group. DFA α2 is a nonlinear index of long-range heart rate dynamics; higher values are thought to reflect greater autonomic homeostasis and regulatory resilience (Peng et al., 1995; Tulppo et al., 2005), states that have been associated with sustained attentional focus and motor stability in precision sports (Ortega and Wang, 2018). The larger reduction in logLF among responders (d = 0.654, *p* = 0.019, uncorrected) is directionally consistent with this pattern, tentatively suggesting a more pronounced attenuation of sympathetic dominance following training in this group; however, given that neither effect survived FDR correction and that the responder subgroup comprised only 4 athletes, these estimates should be regarded as unstable and preliminary. At the behavioral level, responders showed numerically greater improvements in fine aiming control indices, including dwell time within the 10.5-ring and 10.0-ring zones and overall ring score, with large uncorrected effect sizes (d = 1.198–1.370) that did not reach significance after FDR correction. Dwell time within high-precision scoring zones is thought to reflect fine motor control stability during the pre-shot window (Lu et al., 2024); the consistent direction of effect across all three shooting indices may indicate a pattern of practical relevance, though this remains speculative given the small subgroup sizes and should not be interpreted as statistically confirmed. It should be noted that effect-size estimates derived from a subgroup of only four non-responders are likely to be unstable and sensitive to individual data points. Therefore, Cohen’s d values of this magnitude should be interpreted as reflecting the specific composition of the present sample rather than as precise estimates of the population effect. The wide confidence intervals reported in Supplementary Table S3 (e.g., DFA α2: 95% CI [0.178, 3.198]) further illustrate the uncertainty surrounding these estimates. This limitation is also supported by the sensitivity power analysis, which indicates that the present study was underpowered to estimate effects with high precision given the small subgroup size. The four-timepoint DFA α2 trajectories illustrate the descriptive temporal dynamics underlying these between-group patterns, which were not formally tested for statistical differences across timepoints. The divergent pattern observed during the neurofeedback training window (T2 → T3)—an apparent recovery in responders versus a continued decline in non-responders—occurred against a background of comparable baseline values at T1, which is consistent with, but does not establish, the interpretation that this divergence reflects differential responses to the neurofeedback session rather than pre-existing physiological differences. This pattern is broadly consistent with the theoretical framework of neurovisceral integration, which posits that voluntary regulation of cortical activity is coupled with dynamic adjustments in autonomic cardiac control (Thayer and Lane, 2000; Thayer et al., 2009; Skalski et al., 2025), although the present design does not permit a direct test of this mechanism. Related work using synchronized EEG–HRV monitoring in air pistol athletes has reported that the degree of brain–heart co-regulation during the pre-shot window differentiates successful from unsuccessful performance (Wang et al., 2023), which may offer a relevant framework for future hypothesis-driven research in this area. It should also be emphasized that, in the absence of a control or sham-feedback condition, none of the autonomic or performance differences reported above—whether related to DFA α2, logLF, shooting performance indices, or the four-timepoint trajectories—can be attributed specifically to SMR neurofeedback; non-specific factors such as post-exercise recovery, the passage of time between shooting blocks, task-repetition (practice) effects, or general relaxation during the rest periods represent equally plausible explanations for the observed patterns.

No significant between-group differences were observed on any post-training subjective rating dimension—including fatigue, tension, willingness to engage, effort exerted, and perceived difficulty. While this argues against a straightforward explanation of the observed autonomic and performance patterns in terms of differential motivation or effort, the absence of a significant difference in a small, unequal-sized sample should not be over-interpreted as strong evidence of equivalence between groups.

Several limitations should be considered when interpreting the findings of this pilot study. First, the single-group, single-session pre–post design without a control or sham-feedback condition limits causal inference; effects related to time, repeated testing, relaxation, and placebo-like mechanisms cannot be excluded, and all reported associations should therefore be interpreted as correlational rather than causal. This design was adopted primarily to characterize individual differences in SMR self-regulation under real-world constraints on elite athlete availability. Accordingly, the present findings should be regarded as a preliminary step toward, rather than a substitute for, subsequent sham-controlled confirmatory studies. Second, the final analytic sample was small (*N* = 13) and included unequal responder/non-responder subgroups (*n* = 9 vs. *n* = 4), which limited statistical power and the stability of subgroup effect-size estimates. The *a priori* power analysis reported in Section 2.1 was designed specifically for the group-level repeated-measures ANOVA of SMR amplitude and does not extend to the exploratory correlation and subgroup analyses. Therefore, the observed associations (r = 0.50–0.64) and subgroup effects (d = 1.2–1.7) should be interpreted cautiously, as these estimates may be unstable and require replication in larger, adequately powered samples. Third, responders and non-responders were classified *post hoc* based on the observed direction of SMR change during training, which introduces a degree of circularity when this same classification is subsequently used to compare downstream physiological and behavioral outcomes; this should be regarded as a structural limitation of the responder-based analytic approach rather than a finding to be corrected post hoc. Fourth, respiration was not monitored or controlled during data acquisition; athletes breathed naturally throughout the protocol. While this preserved ecological validity, uncontrolled respiration is a known source of variability in frequency-domain HRV indices such as LF and HF power, and future studies may benefit from incorporating paced breathing or respiratory monitoring to reduce this source of noise. Fifth, the single-session design cannot address cumulative training effects or long-term retention of SMR regulatory capacity. Sixth, each subjective dimension was assessed using a single-item rating without systematic psychometric validation, which limits the reliability of these measures. Seventh, given ongoing debate regarding the physiological specificity of frequency-domain HRV indices, including logLF and the LF/HF ratio (Billman, 2013), their interpretation in the present study should remain cautious and should not be taken as definitive evidence of changes in sympathetic or parasympathetic activity. Given these limitations, the present findings are best regarded as an exploratory pilot investigation intended to generate hypotheses for future confirmatory research employing larger samples and appropriate control or sham-feedback conditions.

## 5 Conclusion

In elite rifle athletes, individual SMR self-regulation capacity during a single neurofeedback session was associated with post-training autonomic responses and shooting-performance changes. These preliminary, exploratory findings raise the possibility that SMR amplitude slope may provide a potential indicator of individual differences in neurophysiological responsiveness to neurofeedback. However, given the small sample, absence of a control or sham-feedback condition, and the exploratory nature of the analyses, these findings should not be interpreted as evidence of a causal effect of SMR regulation on autonomic or shooting-performance outcomes. Replication in larger, randomized controlled studies incorporating appropriate control or sham-feedback conditions and repeated training sessions is needed before the utility of SMR slope as an individual-response marker can be established.

## Data Availability

The original contributions presented in the study are included in the article/Supplementary material, further inquiries can be directed to the corresponding author.
