# Supplementary material for: Individual SMR neurofeedback responsiveness during a single session and its associations with autonomic regulation and shooting performance in elite rifle athletes
Source: Front Hum Neurosci. 2026 Sep 4;20:1922593. doi: 10.3389/fnhum.2026.1922593 (PMC13585742; doi:10.3389/fnhum.2026.1922593)
Supplement: Supplementary file 1 [file Table_1.DOCX]

**Supplementary Table S1.** Baseline (T1) comparison of HRV indices between Responders and Non-Responders.

| **Index** | **Unit** | **Responders (n = 9) M ± SD** | **Non-Responders (n = 4) M ± SD** | **t(11)** | **p** | **Cohen's d** |
| --- | --- | --- | --- | --- | --- | --- |
| Stress index | – | 14.65 ± 4.73 | 18.37 ± 7.47 | -1.104 | 0.293 | -0.664 |
| PNS index | – | -1.51 ± 0.73 | -1.66 ± 0.85 | 0.322 | 0.754 | 0.193 |
| SNS index | – | 2.26 ± 1.23 | 3.03 ± 1.75 | -0.928 | 0.373 | -0.558 |
| RMSSD | ms | 25.86 ± 12.11 | 24.35 ± 17.74 | 0.182 | 0.859 | 0.109 |
| SDNN | ms | 34.55 ± 14.17 | 27.03 ± 14.16 | 0.884 | 0.396 | 0.531 |
| *logLF* | log(ms²) | 6.24 ± 0.87 | 5.53 ± 0.97 | 1.315 | 0.215 | 0.790 |
| *logHF* | log(ms²) | 5.58 ± 0.75 | 5.45 ± 1.41 | 0.225 | 0.826 | 0.135 |
| LF (n.u.) | n.u. | 65.34 ± 9.88 | 51.59 ± 18.18 | 1.803 | 0.099 | 1.083 |
| HF (n.u.) | n.u. | 34.65 ± 9.88 | 48.39 ± 18.20 | -1.800 | 0.099 | -1.082 |
| LF/HF | – | 2.13 ± 1.03 | 1.38 ± 1.14 | 1.174 | 0.265 | 0.705 |
| Total Power | ms² | 1185.33 ± 959.33 | 892.17 ± 1013.32 | 0.501 | 0.626 | 0.301 |
| *DFA α1* | – | 1.25 ± 0.19 | 1.12 ± 0.31 | 0.988 | 0.344 | 0.594 |
| *DFA α2* | – | 0.53 ± 0.09 | 0.47 ± 0.07 | 1.126 | 0.284 | 0.677 |

*Note: Values are presented as mean ± standard deviation. Independent-samples t-tests were conducted on all 13 HRV indices recorded at T1 (initial resting baseline) to verify physiological comparability between groups prior to neurofeedback training. No index reached statistical significance (all p > 0.05). PNS, Parasympathetic Nervous System; SNS, Sympathetic Nervous System; RMSSD, root mean square of successive differences; SDNN, standard deviation of normal-to-normal R-R intervals; LF, low frequency; HF, high frequency; n.u., normalized units; DFA, detrended fluctuation analysis (α1: short-term, α2: long-term scaling exponent).*
