# Supplementary material for: Individual SMR neurofeedback responsiveness during a single session and its associations with autonomic regulation and shooting performance in elite rifle athletes
Source: Front Hum Neurosci. 2026 Sep 4;20:1922593. doi: 10.3389/fnhum.2026.1922593 (PMC13585742; doi:10.3389/fnhum.2026.1922593)
Supplement: Supplementary file 2 [file Table_2.DOCX]

**Supplementary Table S2.** Pearson correlations between SMR amplitude slope and all HRV and shooting performance change indices.

| **Variable** | **r** | **95% CI** | **p** | **q (FDR)** | **n** |
| --- | --- | --- | --- | --- | --- |
| ***HRV change indices (Δ = T3 − T2)*** | | | | | |
| ΔPNS index | -0.172 | [-0.661, 0.418] | 0.574 | 0.722 | 13 |
| ΔSNS index | 0.468 | [-0.112, 0.810] | 0.107 | 0.337 | 13 |
| ΔStress index | 0.590 | [0.058, 0.861] | 0.034^*^ | 0.248 | 13 |
| ΔRMSSD | -0.185 | [-0.668, 0.407] | 0.545 | 0.722 | 13 |
| ΔSDNN | -0.469 | [-0.811, 0.110] | 0.106 | 0.337 | 13 |
| ΔlogLF | -0.636 | [-0.879, -0.131] | 0.019^*^ | 0.248 | 13 |
| ΔlogHF | -0.297 | [-0.729, 0.304] | 0.325 | 0.596 | 13 |
| ΔLF (n.u.) | -0.251 | [-0.705, 0.348] | 0.408 | 0.641 | 13 |
| ΔHF (n.u.) | 0.251 | [-0.348, 0.705] | 0.408 | 0.641 | 13 |
| ΔLF/HF | -0.138 | [-0.641, 0.447] | 0.652 | 0.755 | 13 |
| ΔTotal Power | -0.337 | [-0.749, 0.263] | 0.261 | 0.521 | 13 |
| ΔDFA α1 | -0.350 | [-0.756, 0.249] | 0.240 | 0.521 | 13 |
| ΔDFA α2 | 0.509 | [-0.058, 0.828] | 0.076 | 0.337 | 13 |
| ***Shooting performance change indices (Δ = Block 2 − Block 1)*** | | | | | |
| ΔR (shot score) | 0.623 | [0.110, 0.874] | 0.023^*^ | 0.248 | 13 |
| ΔAim time | -0.094 | [-0.613, 0.482] | 0.760 | 0.797 | 13 |
| Δ10.0% | 0.355 | [-0.244, 0.758] | 0.234 | 0.521 | 13 |
| Δ10.5% | 0.500 | [-0.071, 0.824] | 0.082 | 0.337 | 13 |
| Δ10a0% | -0.188 | [-0.670, 0.405] | 0.538 | 0.722 | 13 |
| Δ10a5% | 0.113 | [-0.467, 0.625] | 0.712 | 0.783 | 13 |
| ΔDA | -0.427 | [-0.792, 0.163] | 0.146 | 0.402 | 13 |
| ΔS1 | 0.165 | [-0.425, 0.656] | 0.591 | 0.722 | 13 |
| ΔS2 | 0.034 | [-0.527, 0.574] | 0.913 | 0.913 | 13 |

*Note: Pearson correlations were computed between individual SMR amplitude slope and 13 HRV change indices (Δ = T3 [post-training rest] − T2 [pre-training rest]) and 9 shooting performance change indices (Δ = second 30-shot block − first 30-shot block), for a total of 22 comparisons (N = 13). Benjamini–Hochberg false discovery rate (FDR) correction was applied across all 22 comparisons (q < 0.05). 95% confidence intervals were derived via Fisher’s z-transformation. ^*^ p < 0.05 (uncorrected). Bold q-values indicate q < 0.05 after FDR correction. PNS, Parasympathetic Nervous System; SNS, Sympathetic Nervous System; RMSSD, root mean square of successive differences; SDNN, standard deviation of normal-to-normal R-R intervals; LF, low frequency; HF, high frequency; n.u., normalized units; DFA, detrended fluctuation analysis; R, shot score; S1/S2, aiming point velocity (1-s / 250-ms pre-shot window); DA, distance between mean aiming point and impact point.*
