# Supplementary material for: Individual SMR neurofeedback responsiveness during a single session and its associations with autonomic regulation and shooting performance in elite rifle athletes
Source: Front Hum Neurosci. 2026 Sep 4;20:1922593. doi: 10.3389/fnhum.2026.1922593 (PMC13585742; doi:10.3389/fnhum.2026.1922593)
Supplement: Supplementary file 3 [file Table_3.DOCX]

**Supplementary Table S3.** Between-group comparisons of HRV and shooting performance change indices (Responders vs. Non-Responders).

| **Variable** | **Responders (n = 9) M ± SD** | **Non-Responders (n = 4) M ± SD** | **t(11)** | **p** | **Cohen’s d** | **95% CI** | **q (FDR)** | **n** |
| --- | --- | --- | --- | --- | --- | --- | --- | --- |
| ***HRV change indices (Δ = T3 − T2)*** | | | | | | | | |
| *ΔDFA α2* | 0.13 ± 0.18 | -0.12 ± 0.02 | 2.809 | 0.017^*^ | 1.688 | [0.178, 3.198] | 0.209 | 12 |
| *ΔlogLF* | -0.06 ± 0.48 | 0.74 ± 0.47 | -2.753 | 0.019^*^ | -1.654 | [-3.157, -0.151] | 0.209 | 12 |
| ΔPNS index | 0.14±0.62 | 0.31±0.33 | -0.490 | 0.634 | -0.294 | [-1.472,0.896 ] | 0.880 | 12 |
| ΔSNS index | -0.14±0.57 | -0.56±0.22 | 1.410 | 0.186 | 0.847 | [-0.399,2.058 ] | 0.493 | 12 |
| ΔStress index | -0.35±1.66 | -1.80±2.06 | 1.359 | 0.202 | 0.816 | [-0.426,2.025 ] | 0.493 | 12 |
| ΔRMSSD | 3.21±13.18 | 4.63±4.69 | -0.205 | 0.841 | -0.123 | [-1.299,1.058 ] | 0.925 | 12 |
| ΔSDNN | 2.62±9.89 | 6.62±7.79 | -0.712 | 0.491 | -0.428 | [-1.609,0.773 ] | 0.772 | 12 |
| *ΔlogHF* | 0.06±0.77 | 0.21±0.19 | -0.371 | 0.718 | -0.223 | [-1.399,0.963 ] | 0.880 | 12 |
| ΔHF (n.u.) | 2.48±19.25 | -13.40±12.11 | 1.502 | 0.161 | 0.903 | [-0.351,2.120 ] | 0.493 | 12 |
| ΔLF (n.u.) | -2.50±19.30 | 13.41±12.12 | -1.502 | 0.161 | -0.902 | [-2.119,0.351 ] | 0.493 | 12 |
| ΔTotal Power | 495.77±1620.21 | 823.64±1019.66 | -0.368 | 0.720 | -0.221 | [-1.398,0.965 ] | 0.880 | 12 |
| ΔLF/HF | -0.24±1.30 | 0.33±0.80 | -0.800 | 0.441 | -0.480 | [-1.664,0.724 ] | 0.746 | 12 |
| *ΔDFA α1* | -0.09±0.19 | 0.06±0.23 | -1.223 | 0.247 | -0.735 | [-1.936,0.497 ] | 0.543 | 12 |
| ***Shooting performance change indices (Δ = Block 2 − Block 1)*** | | | | | | | | |
| Δ10.5% | 0.10 ± 0.10 | -0.02 ± 0.04 | 2.279 | 0.044^*^ | 1.369 | [0.038, 2.651] | 0.316 | 12 |
| Δ10.0% | 0.08 ± 0.11 | -0.04 ± 0.04 | 2.078 | 0.062 | 1.249 | [-0.060, 2.511] | 0.316 | 12 |
| ΔR (shot score) | 0.04 ± 0.15 | -0.20 ± 0.29 | 1.993 | 0.072 | 1.198 | [-0.103, 2.452] | 0.316 | 12 |
| ΔAim time | -0.24±1.28 | -0.06 ±1.27 | -0.232 | 0.821 | -0.139 | [-1.315,1.043 ] | 0.925 | 12 |
| Δ10a0% | 0.00 ±0.03 | 0.02 ±0.05 | -0.962 | 0.357 | -0.578 | [-1.767,0.636 ] | 0.655 | 12 |
| Δ10a5% | -0.02± 0.05 | -0.02 ±0.02 | 0.095 | 0.926 | 0.057 | [-1.122,1.234 ] | 0.926 | 12 |
| ΔS1 | -0.08 ±0.89 | -0.16 ±1.56 | 0.122 | 0.905 | 0.073 | [-1.107, 1.250] | 0.926 | 12 |
| ΔS2 | 0.49 ±0.76 | 0.72 ±1.46 | -0.380 | 0.711 | -0.228 | [-1.405,0.958 ] | 0.880 | 12 |
| ΔDA | 0.04±0.25 | 0.25 ±0.43 | -1.137 | 0.280 | -0.683 | [-1.880,0.543 ] | 0.560 | 12 |

*Note: Values are presented as mean ± standard deviation where reported in the manuscript. Independent-samples t-tests were conducted on all 22 change indices (13 HRV indices: Δ = T3 [post-training rest] − T2 [pre-training rest]; 9 shooting performance indices: Δ = second 30-shot block − first 30-shot block). Benjamini–Hochberg false discovery rate (FDR) correction was applied jointly across all 22 comparisons (q < 0.05). 95% confidence intervals are reported for Cohen’s d. – indicates M ± SD not reported in the primary manuscript for that index. ^*^ p < 0.05 (uncorrected). Bold q-values indicate q < 0.05 after FDR correction (none observed). PNS, Parasympathetic Nervous System; SNS, Sympathetic Nervous System; RMSSD, root mean square of successive differences; SDNN, standard deviation of normal-to-normal R-R intervals; LF, low frequency; HF, high frequency; n.u., normalized units; DFA, detrended fluctuation analysis; R, shot score; S1/S2, aiming point velocity; DA, distance between mean aiming point and impact point.*
