# Supplementary material for: Individual SMR neurofeedback responsiveness during a single session and its associations with autonomic regulation and shooting performance in elite rifle athletes
Source: Front Hum Neurosci. 2026 Sep 4;20:1922593. doi: 10.3389/fnhum.2026.1922593 (PMC13585742; doi:10.3389/fnhum.2026.1922593)
Supplement: Supplementary file 4 [file Table_4.DOCX]

**Supplementary Table S4.** Linear mixed-effects model results for four-timepoint (T1–T4) DFA α2 and logLF trajectories, supplementing the change-score analyses in Supplementary Table S3.

***Panel A. Omnibus fixed-effect tests (likelihood ratio χ² tests)***

| **Effect** | **df** | **χ²** | **p** |
| --- | --- | --- | --- |
| *DFA α2* | | | |
| Timepoint | 3 | 3.166 | 0.367 |
| Group | 1 | 0.386 | 0.534 |
| Timepoint × Group | 3 | 11.271 | **0.010*** |
| *logLF* | | | |
| Timepoint | 3 | 24.914 | **<0.001*** |
| Group | 1 | 0.480 | 0.489 |
| Timepoint × Group | 3 | 6.415 | 0.093 |

***Panel B. Planned contrast: differential T2-to-T3 change (Responder vs. Non-Responder)***

| **Variable** | **Estimate** | **SE** | **z** | **p** |
| --- | --- | --- | --- | --- |
| ΔDFA α2 (T3 − T2), Responder vs. Non-Responder | 0.256 | 0.080 | 3.20 | **0.001*** |
| ΔlogLF (T3 − T2), Responder vs. Non-Responder | -0.791 | 0.553 | -1.43 | 0.153 |

***Note:*** *Linear mixed-effects models (LMMs) were fitted for DFA α2 and logLF across all four resting-state timepoints (T1–T4) in JASP (version 0.19.2.0, Mixed Models module), with Timepoint, Group (Responder/Non-Responder), and their interaction specified as fixed effects and a random intercept for participant. A random slope for Timepoint was initially considered but was not estimable given the sample size and was therefore removed from the final model; a random slope for Group was not applicable, as Group does not vary within participants. Fixed effects were evaluated using Type III likelihood-ratio tests (Panel A). To isolate the planned between-group difference in the T2-to-T3 change (Panel B) — a contrast not directly available through JASP's Mixed Models interface — the relevant model coefficients and variance–covariance matrix were exported and the corresponding linear contrast was computed in Python (statsmodels, version 0.14); the resulting estimate was independently verified against the JASP model output. *p < 0.05.*
